# RAGE Modulates Hypoxia/Reoxygenation Injury in Adult Murine Cardiomyocytes via JNK and GSK-3β Signaling Pathways

**DOI:** 10.1371/journal.pone.0010092

**Published:** 2010-04-09

**Authors:** Linshan Shang, Radha Ananthakrishnan, Qing Li, Nosirudeen Quadri, Mariane Abdillahi, Zhengbin Zhu, Wu Qu, Rosa Rosario, Fatouma Touré, Shi Fang Yan, Ann Marie Schmidt, Ravichandran Ramasamy

**Affiliations:** Division of Surgical Science, Department of Surgery, College of Physicians and Surgeons, Columbia University, New York, New York, United States of America; Keio University, Japan

## Abstract

**Background:**

Advanced glycation end-products (AGEs) have been implicated in diverse pathological settings including diabetes, inflammation and acute ischemia/reperfusion injury in the heart. AGEs interact with the receptor for AGEs (RAGE) and transduce signals through activation of MAPKs and proapoptotic pathways. In the current study, adult cardiomyocytes were studied in an *in vitro* ischemia/reperfusion (I/R) injury model to delineate the molecular mechanisms underlying RAGE-mediated injury due to hypoxia/reoxygenation (H/R).

**Methodology/Principal Findings:**

Cardiomyocytes isolated from adult wild-type (WT), homozygous RAGE-null (RKO), and WT mice treated with soluble RAGE (sRAGE) were subjected to hypoxia for 30 minutes alone or followed by reoxygenation for 1 hour. In specific experiments, RAGE ligand carboxymethyllysine (CML)-AGE (termed “CML” in this manuscript) was evaluated *in vitro*. LDH, a marker of cellular injury, was assayed in the supernatant in the presence or absence of signaling inhibitor-treated cardiomyocytes. Cardiomyocyte levels of heterogeneous AGEs were measured using ELISA. A pronounced increase in RAGE expression along with AGEs was observed in H/R vs. normoxia in WT cardiomyocytes. WT cardiomyocytes after H/R displayed increased LDH release compared to RKO or sRAGE-treated cardiomyocytes. Our results revealed significant increases in phospho-JNK in WT cardiomyocytes after H/R. In contrast, neither RKO nor sRAGE-treated cardiomyocytes exhibited increased phosphorylation of JNK after H/R stress. The impact of RAGE deletion on GSK-3β phosphorylation in the cardiomyocytes subjected to H/R revealed significantly higher levels of phospho-GSK-3β/total GSK-3β in RKO, as well as in sRAGE-treated cardiomyocytes versus WT cardiomyocytes after H/R. Further investigation established a key role for Akt, which functions upstream of GSK-3β, in modulating H/R injury in adult cardiomyocytes.

**Conclusions/Significance:**

These data illustrate key roles for RAGE-ligand interaction in the pathogenesis of cardiomyocyte injury induced by hypoxia/reoxygenation and indicate that the effects of RAGE are mediated by JNK activation and dephosphorylation of GSK-3β. The outcome in this study lends further support to the potential use of RAGE blockade as an adjunctive therapy for protection of the ischemic heart.

## Introduction

Mounting evidence indicates that ischemia and ischemia/reperfusion (I/R) play important roles in cardiomyocyte loss in pathophysiological conditions. As the major constituent of myocardium, the ventricular myocyte is a terminally differentiated cell that responds to appropriate external stimuli by adaptive growth. *In vivo*, the myocyte may be exposed to a variety of cellular stresses, such as hypoxia, ischemia, and I/R. Cardiomyocyte loss, both necrotic and apoptotic, is a feature of many pathological conditions in the heart [1]. Because adult cardiomyocytes possess minimal capacity to reenter the cell cycle [2], [3], limiting myocyte loss and protection of the myocyte against injury through suppression of cell death-provoking pathways represents a logical strategy to prevent heart failure [4].

Our understanding of the intracellular signaling pathways that mediate stress responses of the myocardium is evolving. We demonstrated earlier that advanced glycation end-products (AGEs), the products of nonenzymatic glycation and oxidation of proteins and lipids, accumulate in diverse biological settings, such as diabetes, inflammation, and acute I/R in the heart [5], [6], [7]. AGEs interact with the receptor for AGEs (RAGE) which results in the propagation of stress signals and activation of MAPKs, NF-κB, and several proapoptotic pathways [8], [9]. We demonstrated that the specific AGE, carboxymethyllysine (CML), is generated during I/R and that expression of dominant negative signal transduction deficient RAGE in endothelial cells or mononuclear phagocytes attenuates I/R injury in diabetic mice hearts [10]. Also, using pharmacological blockade of the ligand-RAGE interaction and genetic modulation of RAGE, we demonstrated that RAGE-ligand interaction leads to key cell death signaling events in myocardial infarction [11]. In the present study, adult cardiomyocytes were used as an *in vitro* I/R injury model to delineate the molecular mechanisms by which RAGE mediates injury due to hypoxia and reoxygenation. Specifically, the goal was to establish involvement of RAGE in hypoxia/reoxygenation injury in adult cardiomyocytes and establish potential molecular mechanisms by which RAGE-ligand interactions lead to injury. Our results indicate cardiomyocyte RAGE and its ligand CML exert pathogenic effects in these cells and identify JNK and GSK3β signal transduction as key signaling events in adult cardiomyocytes in H/R injury.

## Results

### RAGE and ligands expression increased upon H/R in cardiomyocytes

We employed established methods for isolation of adult cardiomyocytes [12]. These methods yielded at least 70% of the cardiomyocytes displaying rod shape morphology, which was similar to previously reported studies [12]. The identity of our isolated cells as cardiomyocytes was further confirmed by using immunofluorescence and FACS with the cardiomyocyte-specific antibody α-sarcomeric actinin (data not shown).

**Figure 1:**
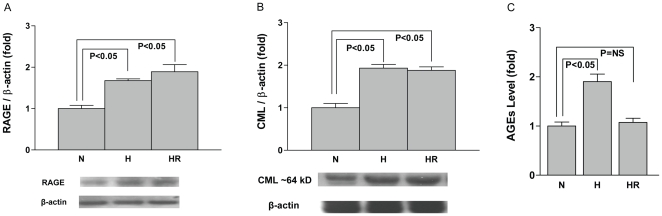
Analysis of receptor for advanced glycation end-products (RAGE) expression and RAGE ligands subjected to hypoxia followed by reoxygenation. WT cardiomyocytes were collected and lysates obtained at the end of normoxia (N), 30 min of hypoxia (H), and hypoxia (30 min) followed by 1 hr reoxygenation (HR), were subjected to Western blot analysis (A–B) and ELISA (C) for the detection of RAGE and its ligands. Cell lysate was probed with (A) anti-RAGE antibody; (B) anti-CML antibody. After being probed with the target antibodies, blots were stripped and reprobed with anti-β-actin IgG. Relative density units are reported. n = 3. (C) 100 µg/well protein was coated and analyzed by ELISA for detection of heterogeneous AGE epitopes. Each sample was measured in two parallel wells and experiment was repeated three times.

To establish a role for RAGE in cardiomyocytes in H/R injury, we first assessed the expression of RAGE in normoxia and H/R conditions in WT cardiomyocytes. Thirty minutes of hypoxia followed by 1 hr of reoxygenation resulted in ≈2.0-fold increase in RAGE expression by Western blotting when compared to cardiomyocytes under normoxia conditions (P<0.05; Fig. 1A). As H/R resulted in increased expression of RAGE in cardiomyocytes, we next sought to identify if H/R resulted in increased generation of RAGE ligand AGEs. Increased detection of CML-AGE was observed after H/R in the cardiomyocytes (≈1.9 fold vs. normoxia, Fig. 1B; P<0.05). Western blotting of the cardiomyocyte lysates revealed three major bands immunoreactive with anti-CML antibody at ∼64 kDa, 47 kDa and 40 kDa. The band at ∼64 kDa was quantified as shown, but similar results were identified for the 47 kDa and 40 kDa bands (not shown). Measurement of heterogeneous AGEs by ELISA revealed a significant increase in AGE levels in cardiomyocytes subjected to hypoxia injury (P<0.05, vs. normoxia; Fig. 1C). These data established that H/R increases RAGE and its ligand AGEs in cardiomyocytes.

### Genetic deletion and pharmacological blockade of RAGE alleviate cellular injury in cardiomyocytes upon H/R

The above experiments strongly pointed to up-regulation of RAGE and its ligand AGEs in H/R. To assess the potential mechanistic involvement of RAGE in cardiomyocytes during H/R injury, cardiomyocytes isolated from WT and RKO mice were subjected to 30 min of hypoxia followed by 1 hr of reoxygenation, and LDH release was measured. Furthermore, sRAGE, a ligand-binding decoy, was administered to mice for 7 days and the cardiomyocytes isolated to test the impact of binding up RAGE ligands and preventing their interaction with RAGE in WT cardiomyocytes subjected to H/R injury.

**Figure 2:**
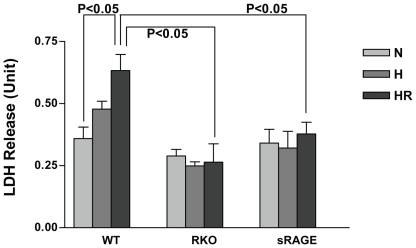
Genetic deletion and pharmacological blockade of RAGE alleviate cellular injury in cardiomyocytes upon H/R. Cardiomyocytes were isolated from WT, RKO, or sRAGE-treated animals and subjected to hypoxia for 30 mins with or without subsequent reoxygenation for 1 hr. The supernatant of cardiomyocytes was collected and the level of LDH released into the medium was determined. n = 6.

LDH release, a marker of cardiomyocyte injury after H/R, was markedly lower in RKO (0.92±0.06 fold that in normoxia) versus wild-type cardiomyocytes (1.81±0.08 fold that in normoxia, p<0.05 for RKO versus WT). Consistent with roles for RAGE ligands, pharmacological blockade of RAGE with sRAGE protected the cardiomyocytes from H/R damage, similar to the effects observed in cardiomyocytes devoid of RAGE (Fig. 2).

### Deletion of RAGE modulates H/R stress through blockade of JNK signaling pathway

**Figure 3:**
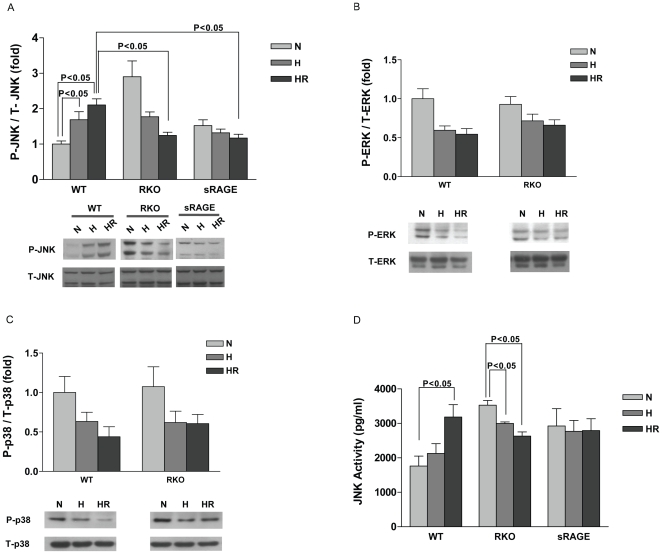
Deletion of RAGE modulates H/R stress via blockade of JNK signaling pathway. Cardiomyocytes were collected and lysates obtained at the end of normoxia (N), 30 min of hypoxia (H), and hypoxia (30 min) followed by 1 hr reoxygenation (HR). Lysates were subjected to Western blot analysis for the detection of (A) phospho-JNK and total-JNK. (B) phospho/total-ERK and (C) phospho/total-p38 level. n = 5. (D) JNK activity was measured by using commercially available ELISA kit; n = 3.

In view of the significant impact of deletion of RAGE on H/R injury in cardiomyocytes, we next sought to examine the potential impact of RAGE on the major early signal transduction mechanisms linked to the recruitment of cell death pathways. Since our previous work in the whole heart consequent to infarction of the left anterior descending coronary artery demonstrated that JNK signal transduction is a central downstream effector pathway of the ligand-RAGE axis during ischemia/reperfusion, we sought to determine the effect of this pathway in isolated adult cardiomyocytes exposed to H/R. Significant increases in phospho-JNK were observed following H/R in WT cardiomyocytes (comparing WT normoxia vs. WT H/R). In contrast, RKO and sRAGE-treated cardiomyocytes did not reveal increases in phospho-JNK levels after H/R, although basal levels of phospho-JNK were consistently higher in RKO vs. WT cardiomyocytes (P<0.05, Fig. 3A and B). Note that in these studies, activity of JNK was measured both by Western blotting and by direct activity assay (Fig. 3A and D, respectively).

In addition to the JNK pathway, we also investigated changes in p38 and ERK kinases that have been shown to be central players in myocardial ischemia-reperfusion injury. As shown in Figure 3B and C, both WT and RKO cardiomyocytes showed similar changes in p38 and ERK during H/R stress despite significant degrees of injury in H/R as illustrated above.

**Figure 4:**
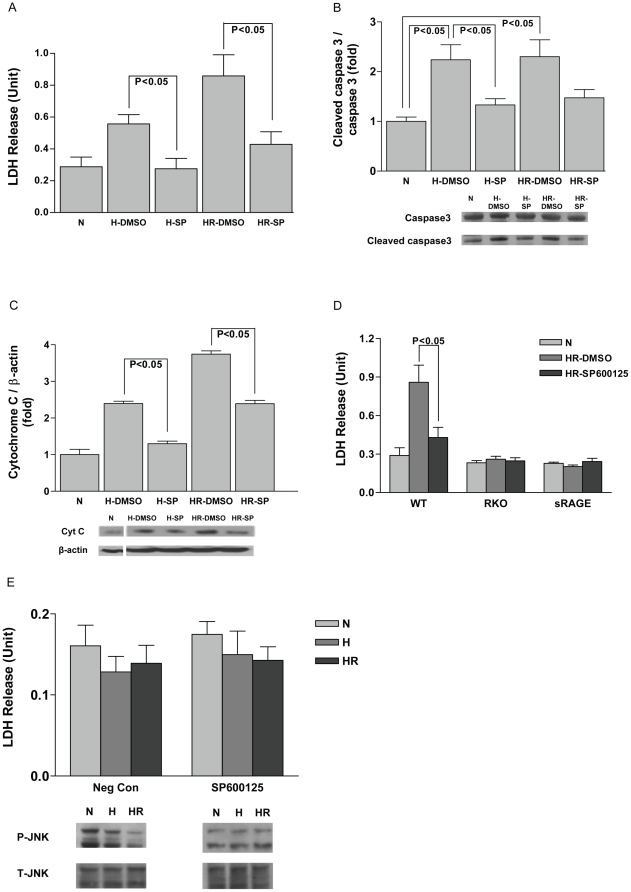
Inhibition of JNK alleviates the injury due to H/R in cardiomyocytes. WT cardiomyocytes were subjected to hypoxia/reoxygenation after 1 hr incubation with JNK inhibitor SP600125 (10 µM) or the vehicle control DMSO. (A) Cell supernatant was collected for LDH level measurement. n = 3. (B) Cleaved caspase 3 levels and (C) Cytochrome c were detected after hypoxia/reoxygenation by Western blot analysis. Data are representative of three independent experiments. (D) Cardiomyocytes isolated from WT and RKO and sRAGE-treated mice were incubated with JNK inhibitor SP600125 (10 µM) or its vehicle control DMSO for 1 hr, followed by hypoxia 30 mins/reoxygenation 1 hr treatment. Cell supernatant was collected for LDH release measurement. (E) The hearts of RKO mice were pre-perfused with JNK-specific inhibitor SP600125 or its negative control for 30 mins prior to the cardiomyocyte isolation process. Cell supernatant was collected for LDH level measurement. Pre-perfusion with the JNK inhibitor did not abrogate the protective effects of RAGE deletion in H/R. n = 3. SP: SP600125.

To further establish the importance of JNK in mediating injury due to H/R in cardiomyocytes, the JNK-specific inhibitor SP600125 was used. As shown in Fig. 4A, inhibition of the JNK signaling pathway attenuated injury due to hypoxia or H/R in WT cardiomyocytes, as assessed by release of LDH. Furthermore, attenuation of increases in cleaved caspase 3 and cytochrome c release was observed in WT cardiomyocytes treated with the JNK inhibitor (Fig. 4B and C). Studies investigating the effects of JNK signaling blockade in RKO and sRAGE-treated cardiomyocytes using SP600125 indicated no additional protective effect on release of LDH (Fig. 4D), suggesting that RAGE deletion fully blocks the effects of JNK signaling in cardiomyocytes under H/R conditions.

To further probe the implications of the apparently high basal level of phospho-JNK in normoxic cardiomyocytes, we performed additional studies. We pre-perfused the hearts of RKO mice with JNK-specific inhibitor SP600125 and its negative control for 30 mins before the cardiomyocyte isolation process. LDH measurement for validating cell injury extent showed that pre-inhibition of JNK activation in the RKO heart did not eliminate the protective effects of RAGE deletion under H/R injury (Fig. 4E).

### Deletion of RAGE promotes cardiomyocyte survival through enhancing the phosphorylation of GSK-3β

**Figure 5:**
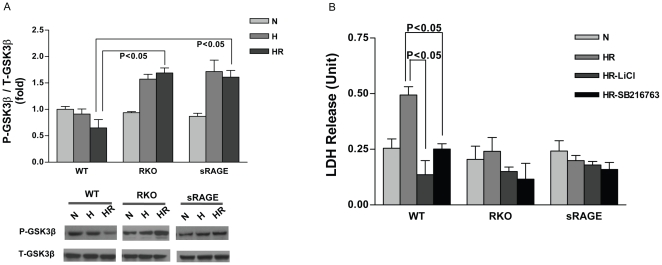
Deletion of RAGE promotes cardiomyocyte survival in H/R by enhancing phosphorylation of GSK3β. (A) Cells were collected and lysates obtained at the end of normoxia (N), 30 mins of hypoxia (H), and hypoxia (30 min) followed by 1 hr reoxygenation (HR). Lysates from WT, RKO and sRAGE-treated cardiomyocytes were subjected to Western blot analysis for the detection of phospho-S9-GSK3β and total-GSK3β. n = 3. (B) Cardiomyocytes were incubated with or without GSK3 inhibitors LiCl (12.5 mM) and SB216763 (3 µM) for 1 hr, followed by 30 min hypoxia/1 hr reoxygenation (HR), cell supernatant was collected for LDH release measurement. n = 3.

Since several studies suggest a crucial role for GSK-3β in protecting hearts during ischemia/reperfusion, we investigated the impact of RAGE deletion on GSK-3β phosphorylation in cardiomyocytes subjected to H/R stress. We first explored levels of GSK-3β and its phosphorylated form in H/R-treated cardiomyocytes from WT, RKO and sRAGE-treated animals. Significantly higher levels of phospho-GSK-3β/total GSK-3β were noted in RKO, as well as sRAGE-treated mice versus WT cardiomyocytes after H/R (P<0.05; Fig. 5A).

To determine if deletion of RAGE protects cardiomyocytes from H/R injury via increases in phosphorylation of GSK-3β, two different types of GSK-3 inhibitors were used in our studies. Cardiomyocytes were treated with (a) Lithium Chloride (LiCl), which targets the inhibitory phosphorylation site of GSK3α-Ser21/GSK3β-Ser9; and (b) SB216763, which targets the stimulatory phosphorylation site GSK3α-Tyr279/GSK3β-Tyr216. Significant attenuation of H/R-induced LDH release was observed in WT cardiomyocytes incubated with both inhibitors (Fig. 5B). Since the endogenous level of GSK-3 is highly abundant, we explored if administration of GSK-3 inhibitors LiCl and SB216763 to RKO cardiomyocytes affords additional protection during H/R stress. The administration of GSK3 signaling inhibitors did not exert additional beneficial effect in the cardiomyocytes isolated from RKO and sRAGE-treated mice as assessed by LDH release (Fig. 5B).

**Figure 6:**
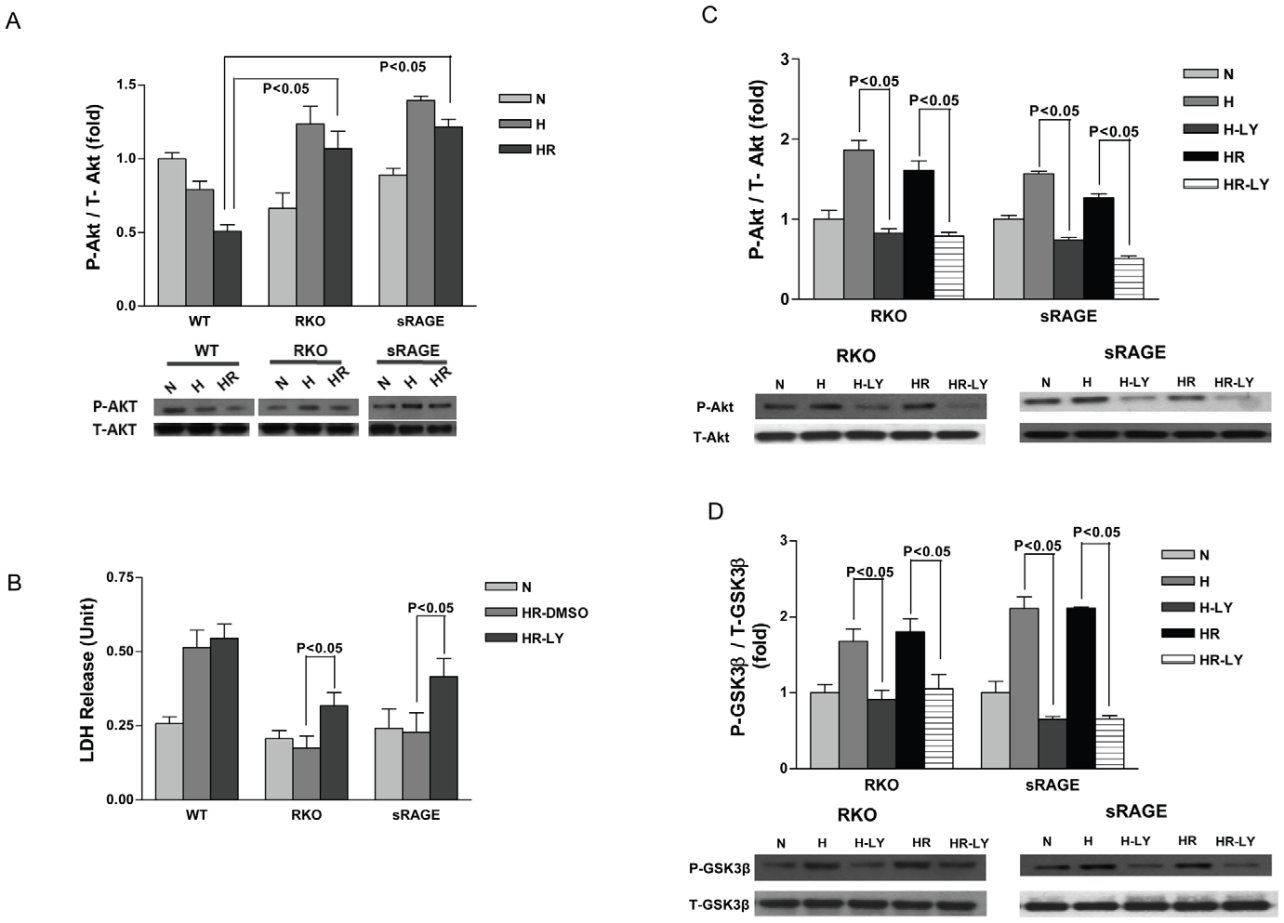
Akt/PKB contributes to the phosphorylation of GSK. (A) Cells were collected and lysates obtained at the end of normoxia (N), 30 min of hypoxia (H), hypoxia (30 min) followed by 1 hr reoxygenation (HR). Lysates from WT, RKO and sRAGE-treated cardiomyocytes subjected to Western blot analysis for the detection of phospho-Akt and total-Akt. n = 3. (B) Cardiomyocytes were incubated with LY294002 (10 µM) or its vehicle control DMSO for 1 hr, followed by HR. Cell supernatant was collected for LDH release measurement. n = 3. (C–D) Cardiomyocytes were incubated with LY294002 (10 µM) or its vehicle control DMSO for 1 hr, followed by HR. Cell lysate was collected for Western blot studies for detection of phospho/total Akt (C) and GSK3β (D). n = 3. LY: LY294002.

Since PI3K/Akt is a well-established upstream kinase that has been shown to phosphorylate GSK-3 at serine residues, we investigated Akt phosphorylation patterns in WT, RKO and sRAGE-treated cardiomyocytes upon H/R. As shown in Fig. 6A, phospho-Akt in WT cardiomyocytes was significantly lower than that observed in RKO and sRAGE-treated cardiomyocytes during H/R (p<0.05). To establish if Akt phosphorylation is a key event that modulates H/R injury, WT and RKO as well as sRAGE-treated cardiomyocytes were treated with the upstream phosphoinositol-3 kinase (PI3-K) signaling inhibitor LY249002 during H/R. As shown in Fig. 6B, LDH release was marginally impacted in WT cardiomyocytes subjected to H/R stress in the presence and absence of LY249002, whereas in RKO and sRAGE-treated cardiomyocytes a significant increase in LDH release was observed in LY249002-treated cells *vs.* vehicle treatment. Although PI3-K inhibition in RKO increased LDH release, the magnitude of increases in injury was still lower than that in WT cardiomyocytes subjected to H/R.

To further explore the signaling cascade involving Akt and GSK phosphorylation, the level of p-GSK was studied with the addition of LY249002. As shown in figure 6C and 6D, changes in phospho-Akt phosphorylation correlated with modulation of phospho-GSK, suggesting that Akt was upstream of GSK-3β. These data indicate that PI3-K-Akt-GSK-3β is a central signaling mechanism by which RAGE deletion exerts protective effects on cardiomyocytes during H/R.

### Assessment of potential interaction between JNK and Akt signaling pathways in the cardiomyocytes in H/R

**Figure 7:**
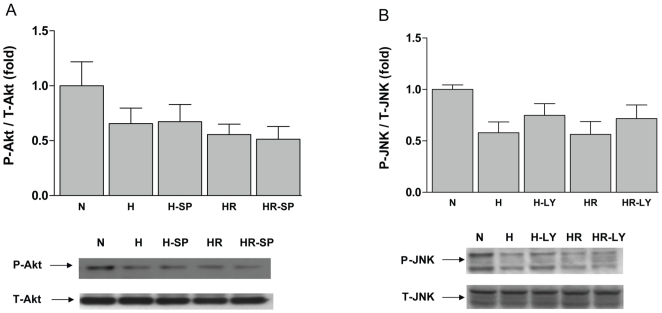
Signaling pathways cross-talk study. (A) Cardiomyocytes isolated from WT mice were incubated with SP600125 (10 µM) or its vehicle control DMSO for 1 hr, followed by 30 min of hypoxia (H), hypoxia (30 min) and 1 hr reoxygenation (HR). Cell lysates were collected for the study of phospho/total-Akt by Western blot. n = 3. (B) Cardiomyocytes isolated from RKO mice were incubated with LY294002 (10 µM) or its vehicle control DMSO for 1 hr, followed by 30 min of hypoxia (H), hypoxia (30 min) and 1 hr reoxygenation (HR). Cell lysates were collected for the study of phospho/total-JNK by western blot. n = 3. SP: SP600125; LY: LY294002.

To further investigate the possibility of cross-talk between these two signaling pathways, JNK and Akt, studies were carried out to determine the activation of either of the kinases in the presence of respective inhibitors. As shown in figure 7A and B, inhibition of JNK kinase with SP600125 in WT cardiomyocytes did not have any apparent effect on Akt phosphorylation. Similarly, inhibition of Akt in RKO cardiomyocytes did not alter JNK phosphorylation significantly.

### Impacts of RAGE ligand on modulating H/R stress signaling machinery in the cardiomyocytes

**Figure 8:**
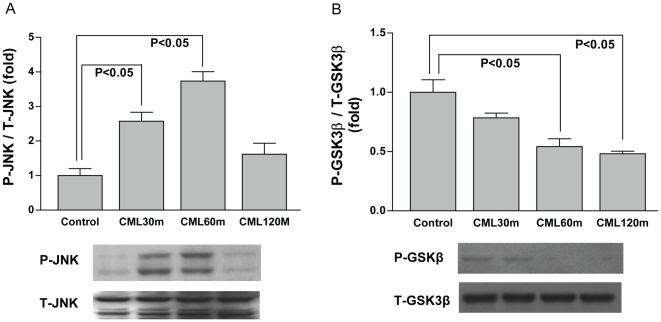
Impact of RAGE ligand on key stress signaling pathways in the absence of H/R in cardiomyocytes. WT cardiomyocytes were incubated with CML-AGE (50 µg/ml) for different time points as indicated. Cells were lysed and subjected to Western blot analysis for the detection of phospho-JNK and total-JNK level (A), as well as phospho-S9-GSK3β and total- GSK3β (B). Data are representative of three independent experiments.

Lastly, as our data revealed increases in RAGE ligand CML-AGE during H/R, we sought to test if exogenous treatment of WT cardiomyocytes with the RAGE ligand CML without H/R would modulate phosphorylation of JNK and GSK3β pathways. As shown in Figure 8A and B, incubation of WT cardiomyocytes with CML significantly increased the phosphorylation of JNK, and suppressed phosphorylation of GSK3β in a time- dependent manner.

## Discussion

Our earlier studies demonstrated that induction of I/R in the intact murine heart resulted in significant upregulation of RAGE, particularly in cardiomyocytes. Further, we reported that blockade of RAGE or genetic deletion of RAGE protects the myocardium from ischemia/reperfusion injury in a murine model. Thus, the current study was designed to elucidate the mechanism by which RAGE induced hypoxia/reoxygenation injury in adult cardiomyocytes. We report that analogous to experiments in I/R in the intact heart, H/R induces significant increases in expression of RAGE and its ligands in cardiomyocytes. Further studies on elucidating the potential signal transduction pathways revealed that JNK signaling was remarkably down-regulated in RKO cardiomyocytes, and that phosphorylation of GSK-3β was significantly increased in RKO cardiomyocytes. Pharmacological blockade of RAGE using the ligand binding decoy sRAGE confirmed that RAGE mediates some of its effects via JNK and GSK-3β.

Our previous data revealed that LAD occlusion itself produced the pre-AGE methylglyoxal (MG), followed by significant AGE production during reperfusion [11]. In isolated perfused heart studies, it was shown that ischemia/reperfusion generates CML and that its interaction with RAGE is a key mediator of injury [10]. Since multiple cell types express RAGE, contributions of RAGE to LAD or I/R injury may be evoked from multiple cellular sources both innate and exogenous to the heart. In the present study, we show using genetic and pharmacological approaches that upregulation of RAGE and its ligands such as CML-AGE is linked to H/R injury in cardiomyocytes. These data are consistent with an important role for cardiomyocyte RAGE in mediating injury due to LAD occlusion/reperfusion in the *in vivo* heart.

Cardioprotection rendered by JNK inhibition has been supported by a number of studies in the literature [13], [14], [15], [16]. However, some studies have suggested that JNKs are capable of transducing antiapoptotic signals, but the mechanisms of these prosurvival effects are much less clear than the mechanisms promoting cell death [17], [18], [19], [20]. Kaiser *et al*. showed that JNK1−/−, JNK2−/−, and transgenic mice expressing dominant negative JNK1/2 within the heart have less JNK activity in the heart, and less injury and cellular apoptosis *in vivo* following ischemia/reperfusion injury. In contrast, MKK7 transgenic mice, which are associated with a mild increase in JNK1/2 activity, also promoted cellular protection following I/R injury *in vivo* [21]. These dichotomous reports about JNK effects likely reflect differences in the contribution of specific cell types towards injury. In cardiomyocytes, expression of dominant negative JNK1 or dominant-negative MKK4 increased nitric oxide-induced cardiomyocytes apoptosis, suggesting a protective role for early, transient activation of JNK signaling [13]. In cultured neonatal cardiomyocytes, oxidative and hypoxic stress-mediated injury was increased by expression of JNK inhibitory mutants [22]. Shao *et al*. found inhibition of JNK activity leads to increased apoptosis in cultured neonatal cardiomyocytes and increased infarct size in *vivo* [23]. Consistent with our previous observation, in contrast to WT cardiomyocytes, RKO cardiomyocytes showed significantly decreased JNK activity upon H/R injury. After pre-incubation with a JNK specific inhibitor, WT cardiomyocytes exhibited reduced cell damage after H/R injury. These data provide strong support for the premise that RAGE mediates H/R injury, in part via modulation of JNK phosphorylation in cardiomyocytes. The fact that our studies *in vivo* [11] agree closely with the results of our present experiments in isolated adult cardiomyocytes strengthens our conclusion of the potential cardioprotective role played by RAGE ablation in JNK inhibition. One intriguing observation in the present study is that compared to WT cardiomyocytes, a higher basal level (under the nomoxia condition) of JNK activity was observed in isolated RKO cardiomyocytes. The reasons underlying higher baseline phospho-JNK in RKO in normoxia are unclear, however, our data indicated that after H/R, phosphorylation of JNK was not increased in the RKO cardiomyocytes, unlike that observed in the WT cardiomyocytes. It is possible that kinases such as AMPK may play a role in basal levels of phosphorylated JNK in these cells [24]. Further study of pre-perfusing the RKO hearts with JNK-specific inhibitor prior to the isolation process showed that pre-inhibition of JNK activation in these hearts did not abrogate the protective effects of RAGE deletion seen in H/R-stressed cardiomyocytes. Taken together, our data indicate that inhibition of JNK in RKO cardiomyocytes post H/R leads to protection; and pre-ischemic JNK activity has no important role in the protection seen in RKO cardiomyocytes.

There are two GSK3 isoforms, α and β (51 and 47 kDa, respectively); the functional effects of GSK3β have been especially highlighted in the heart. GSK3αβ is an essential signaling kinase for many physiological processes, including insulin action, energy metabolism, circadian rhythm, and neuroprotection [25]. At the cellular level, GSK3 regulates cell proliferation, differentiation, and death. A number of transcription factors have been identified as substrates of GSK-3, such as c-Jun, CREB, NFATs, and C/EBPs [26], [27], [28], [29]. Phosphorylation of these transcription factors mediates cellular and physiological functions of GSK3 [30]. Pharmacological inhibition of GSK3β reduced infarct size and improved post-ischemic function [31]–[32]. Consistent with the cardioprotection achieved by inhibiting the activity of GSK3β, the cardiomyocytes of RKO mice showed significantly enhanced phospho-GSK upon H/R injury.

Unlike many signaling kinases, GSK3 is constitutively active and inhibition of GSK3 activity leads to signaling propagation. Several upstream regulators have been reported to turn off the activity of GSK3, among which Akt/PKB is a well-characterized Ser/Thr kinase phosphorylating GSK3. Akt/PKB phosphorylates GSK3α(Ser-21) and GSK3β(Ser-9), and these residues lie in a typical Akt/PKB consensus substrate motif [33]. However, GSK3α(Ser-21)/GSK3β(Ser-9) are phosphorylated by other kinases which also recognize the “Akt/PKB consensus sequence.” Markou *et al* demonstrated inhibition of GSK3β as a consequence of its phosphorylation by Akt/PKB in cardiomyocytes [34]. Akt, a central regulator of cardiomyocyte survival, has been found to protect cardiomyocytes against ischemia/reperfusion injury in the mouse heart [35]. Consistent with these studies, our data show increased phosphorylation of Akt and GSK3β and reduced injury due to H/R stress and provide direct evidence, in RKO cardiomyocytes, for the pivotal role of RAGE in this process. Further, our studies with inhibitors revealed no significant cross-talk between the two potential signaling pathways, JNK and Akt, in adult cardiomyocytes subjected to H/R injury.

Taken together, these data illustrate key roles for RAGE-ligand interaction in the pathogenesis of cardiomyocyte injury induced by hypoxia and reoxygenation and that the effects of RAGE are mediated by JNK activation and dephosphorylation of GSK3β. The outcome in this study lends further support to the potential use of RAGE blockade as an adjunctive therapy for protection of the ischemic heart.

## Materials and Methods

### Animals

All animal experiments were approved by the Institutional Animal Care and Use Committee of Columbia University and conformed to the guidelines outlined in the National Institutes of Health Guide for Care and Use of Laboratory Animals (NIH Pub. No. 85-23, 1996). Male C57BL/6 mice were purchased from The Jackson Laboratory (Bar Harbor, ME) and were used as control wild-type (WT) mice. Homozygous RAGE-null (RKO) mice (generated in the 129 strain) were backcrossed for >12 generations into C57BL/6 mice; homozygous RKO animals were used in the experiments for comparisons with WT mice. Male mice weighing 25–30 g at age 10–12 weeks were used in all experiments and maintained in a temperature-controlled room with alternating 12:12-h light-dark cycles. Soluble RAGE (sRAGE) at 100 µg/day or equal volumes of its diluent, PBS (vehicle), was administered by an intraperitoneal route for 7 days and the mice were sacrificed 1 hour after the last treatment and the adult cardiomyocytes were immediately isolated. For JNK inhibition studies, the hearts of RKO mice were pre-perfused with JNK-specific inhibitor SP600125 or its negative control for 30 mins prior to the cardiomyocyte isolation process.

### Reagents

The primary antibodies used were anti-mouse/rat RAGE (Gene Tex Inc.), anti-CML monoclonal antibody (COSMO Bio CO., LTD); anti-phospho-JNK antibody (Promega); total-JNK antibody, anti-phospho-ERK/total-ERK antibody, anti-phospho-p38/total-p38 antibody, anti-phospho-GSK3β/totalGSK3β, anti-phospho-Akt (Thr308 and Ser473)/total-Akt IgG, anti-caspase-3 IgG (Cell Signaling); anti-cytochrome c IgG (BD Pharmingen); and anti-beta-actin IgG (BD Biosciences Pharmingen). The secondary antibodies used were goat-anti-rabbit IgG-peroxidase antibody and rabbit-anti-mouse IgG-peroxidase antibody (Sigma). All primary antibodies were diluted 1∶1000 prior to use in western blot studies. PI3-K inhibitor LY294002 and JNK inhibitor SP600125 as well as negative control were purchased from Calbiochem. GSK3 inhibitor Lithium Chloride and SB216763 were obtained from Fluka and Sigma, respectively.

### Isolation of adult ventricular cardiomyocytes

Myocytes were isolated from untreated or sRAGE treated adult mice hearts by a modified method described earlier [12]. In brief, hearts excised from anesthetized mice were subjected to Langendorff perfusion with Krebs medium containing calcium (2.5 mM) for 5 minutes, followed by perfusion with Calcium free Krebs medium (8–10 minutes). Hearts were then perfused with an enzymatic solution containing collagenase type II (0.35 mg/ml; Worthington, Freehold, NJ) and protease type XIV (0.01 mg/ml, Sigma) for 5–8 minutes. 50 µM Ca^2+^ was then added into the enzyme solution for perfusing the heart for another 5–10 minutes until the hearts became soft. The hearts were then removed, minced into small pieces and were subjected to further serial enzymatic digestion at 37°C for 1–3 minutes. Cardiomyocytes in the digests were collected by centrifugation at 500 rpm for a minute and the myocyte pellet was resuspended in storage medium (Ca^2+^ free Kreb's containing 1% BSA and Calcium (125 µM). Extracellular Ca^2+^ was incrementally added back to 500 µM over a period of 40 minutes. The rod shaped myocytes settled down immediately, whereas round ones and other cells experienced extended floating in the supernatant. By aspirating the supernatant and repeated washing, we obtained rod-shaped myocyte population. Cardiomyocytes were incubated in Kreb's buffer containing 1 mM Ca^2+^ for the following study. Cell viability was assessed by using the commercially available CellTiter-FluorTM kit (Promega, Madison WI, USA). Characterization of cardiomyocytes was done by staining with α-sarcomeric actinin antibody (Sigma, St. Louis, MO, USA) and FACS study. Isolated cardiomyocytes were subjected to hypoxic stress for 30 minutes under 0.5% oxygen using an In Vivo 400 hypoxic workstation maintained at 37°C followed with or without reoxygenation for 1 hr. In specific experiments, cardiomyocytes were treated with SP600125 (10 µM), LY294002 (10 µM), LiCl (12.5 mM), SB216763 (3 µM) for 60 minutes prior to hypoxia/reoxygenation. In experiments involving specific RAGE ligand CML-AGE, myocytes were incubated in vitro with CML-AGE (50 µg/ml) for various times ranging from 0 to 2 hrs.

### LDH release

Cardiomyocyte injury due to H/R stress was assessed by measuring LDH release in the supernatants that were collected at the end of hypoxia, or H/R. LDH was measured using the commercially available kit (Pointe scientific, Inc.) as published earlier [10].

### Western blot analysis

Lysates from cardiomyocytes, subjected to normoxia, hypoxia, hypoxia/reoxygenation stress and other treatments, were obtained using commercially available kits which contained the protease and phosphatase inhibitors (PIERCE). The protein concentration was determined using a DC Protein Assay kit (Bio-Rad). Equal amounts of protein were separated by SDS-PAGE (4–12% gradient gels), and proteins were transferred to a nitrocellulose membrane (Invitrogen). After blocking in 5% dry milk in TBST (20 mM Tris-HCI, pH 7.5, 250 mM NaCl, 0.1% Tween 20), membranes were incubated overnight with target primary antibodies (1∶1000 dilution) according to the manufacturer's instructions. Membranes were incubated sequentially with secondary antibody for 1 hr. Blots were visualized with an ECL Horseradish Peroxidase Western Blot Detection System (Cell Signaling), and quantitative analysis was performed using Image Quant TL software (Amersham).

### ELISA assay of AGEs

Isolated adult cardiomyocytes were subjected to hypoxia and reoxygenation procedures and cell lysates were prepared as described above. 100 µg/well protein was coated overnight onto an ELISA 96-well plate using carbonate-bicarbonate buffer (Sigma). AGE ELISA was performed using T-gel (Pierce) affinity-purified chicken anti-AGE as the primary antibody, at a concentration of 30 µg/mL for 3 hours at room temperature. The secondary antibody (anti-chicken IgY) (Sigma) was diluted to 1∶10 000 for 1 hour at room temperature. The signals were developed in phosphate-citrate (Sigma) and hydrogen peroxide (Sigma). Ribose glycated albumin was used to prepare the standard curve. Each sample was measured in two parallel wells and experiment was repeated three times.

### ELISA assay of JNK activity

Detection of phospho-JNK was carried out by using a commercially available ELISA kit (SA Biosciences, Catalog No. 900-106), according to the manufacture's instruction.

### Statistical analysis

All data are reported as numbers of experiments or samples (n) and means±SD for each experiment. Student's t-test was used to compare two groups. A probability value of P<0.05 indicated statistical significance.

## References

[1] Kajstura J, Cheng W, Reiss K, Clark WA, Sonnenblick EH (1996). Apoptotic and necrotic myocyte cell deaths are independent contributing variables of infarct size in rats.. Lab Invest.

[2] Soonpaa MH, Field LJ (1998). Survey of studies examining mammalian cardiomyocyte DNA synthesis.. Circ Res.

[3] Anversa P, Kajstura J (1998). Ventricular myocytes are not terminally differentiated in the adult mammalian heart.. Circ Res.

[4] MacLellan WR, Schneider MD (1997). Death by design. Programmed cell death in cardiovascular biology and disease.. Circ Res.

[5] Bucciarelli LG, Kaneko M, Ananthakrishnan R, Harja E, Lee LK (2006). Receptor for advanced-glycation end products: key modulator of myocardial ischemic injury.. Circulation.

[6] Park L, Raman KG, Lee KJ, Lu Y, Ferran LJ (1998). Suppression of accelerated diabetic atherosclerosis by the soluble receptor for advanced glycation endproducts.. Nat Med.

[7] Rong LL, Yan SF, Wendt T, Hans D, Pachydaki S (2004). RAGE modulates peripheral nerve regeneration via recruitment of both inflammatory and axonal outgrowth pathways.. FASEB J.

[8] Cataldegirmen G, Zeng S, Feirt N, Ippagunta N, Dun H (2005). RAGE limits regeneration after massive liver injury by coordinated suppression of TNF-alpha and NF-kappaB.. J Exp Med.

[9] Sakaguchi T, Yan SF, Yan SD, Belov D, Rong LL (2003). Central role of RAGE-dependent neointimal expansion in arterial restenosis.. J Clin Invest.

[10] Bucciarelli LG, Ananthakrishnan R, Hwang YC, Kaneko M, Song F (2008). RAGE and modulation of ischemic injury in the diabetic myocardium.. Diabetes.

[11] Aleshin A, Ananthakrishnan R, Li Q, Rosario R, Lu Y (2008). RAGE modulates myocardial injury consequent to LAD infarction via impact on JNK and STAT signaling in a murine model.. Am J Physiol Heart Circ Physiol.

[12] Das A, Xi L, Kukreja RC (2005). Phosphodiesterase-5 inhibitor sildenafil preconditions adult cardiac myocytes against necrosis and apoptosis. Essential role of nitric oxide signaling.. J Biol Chem.

[13] Andreka P, Zang J, Dougherty C, Slepak TI, Webster KA (2001). Cytoprotection by Jun kinase during nitric oxide-induced cardiac myocyte apoptosis.. Circ Res.

[14] Cicconi S, Ventura N, Pastore D, Bonini P, Di Nardo P (2003). Characterization of apoptosis signal transduction pathways in HL-5 cardiomyocytes exposed to ischemia/reperfusion oxidative stress model.. J Cell Physiol.

[15] Minamino T, Yujiri T, Papst PJ, Chan ED, Johnson GL (1999). MEKK1 suppresses oxidative stress-induced apoptosis of embryonic stem cell-derived cardiac myocytes.. Proc Natl Acad Sci U S A.

[16] Dougherty CJ, Kubasiak LA, Frazier DP, Li H, Xiong WC (2004). Mitochondrial signals initiate the activation of c-Jun N-terminal kinase (JNK) by hypoxia-reoxygenation.. FASEB J.

[17] Aoki H, Kang PM, Hampe J, Yoshimura K, Noma T (2002). Direct activation of mitochondrial apoptosis machinery by c-Jun N-terminal kinase in adult cardiac myocytes.. J Biol Chem.

[18] He H, Li HL, Lin A, Gottlieb RA (1999). Activation of the JNK pathway is important for cardiomyocyte death in response to simulated ischemia.. Cell Death Differ.

[19] Hreniuk D, Garay M, Gaarde W, Monia BP, McKay RA (2001). Inhibition of c-Jun N-terminal kinase 1, but not c-Jun N-terminal kinase 2, suppresses apoptosis induced by ischemia/reoxygenation in rat cardiac myocytes.. Mol Pharmacol.

[20] Kumar Y, Tatu U (2003). Stress protein flux during recovery from simulated ischemia: induced heat shock protein 70 confers cytoprotection by suppressing JNK activation and inhibiting apoptotic cell death.. Proteomics.

[21] Kaiser RA, Liang Q, Bueno O, Huang Y, Lackey T (2005). Genetic inhibition or activation of JNK1/2 protects the myocardium from ischemia-reperfusion-induced cell death in vivo.. J Biol Chem.

[22] Dougherty CJ, Kubasiak LA, Prentice H, Andreka P, Bishopric NH (2002). Activation of c-Jun N-terminal kinase promotes survival of cardiac myocytes after oxidative stress.. Biochem J.

[23] Shao Z, Bhattacharya K, Hsich E, Park L, Walters B (2006). c-Jun N-terminal kinases mediate reactivation of Akt and cardiomyocyte survival after hypoxic injury in vitro and in vivo.. Circ Res.

[24] Yun H, Kim HS, Lee S, Kang I, Kim SS (2009). AMP kinase signaling determines whether c-Jun N-terminal kinase promotes survival or apoptosis during glucose deprivation.. Carcinogenesis.

[25] Gould TD, Manji HK (2005). Glycogen synthase kinase-3: a putative molecular target for lithium mimetic drugs.. Neuropsychopharmacology.

[26] Doble BW, Woodgett JR (2003). GSK-3: tricks of the trade for a multi-tasking kinase.. J Cell Sci.

[27] Kockeritz L, Doble B, Patel S, Woodgett JR (2006). Glycogen synthase kinase-3–an overview of an over-achieving protein kinase.. Curr Drug Targets.

[28] Woodgett JR (2001). Judging a protein by more than its name: GSK-3.. Sci STKE.

[29] Coghlan MP, Culbert AA, Cross DA, Corcoran SL, Yates JW (2000). Selective small molecule inhibitors of glycogen synthase kinase-3 modulate glycogen metabolism and gene transcription.. Chem Biol.

[30] Grimes CA, Jope RS (2001). CREB DNA binding activity is inhibited by glycogen synthase kinase-3 beta and facilitated by lithium.. J Neurochem.

[31] Tong H, Imahashi K, Steenbergen C, Murphy E (2002). Phosphorylation of glycogen synthase kinase-3beta during preconditioning through a phosphatidylinositol-3-kinase–dependent pathway is cardioprotective.. Circ Res.

[32] Juhaszova M, Zorov DB, Kim SH, Pepe S, Fu Q (2004). Glycogen synthase kinase-3beta mediates convergence of protection signaling to inhibit the mitochondrial permeability transition pore.. J Clin Invest.

[33] Obata T, Yaffe MB, Leparc GG, Piro ET, Maegawa H (2000). Peptide and protein library screening defines optimal substrate motifs for AKT/PKB.. J Biol Chem.

[34] Markou T, Cullingford TE, Giraldo A, Weiss SC, Alsafi A (2008). Glycogen synthase kinases 3alpha and 3beta in cardiac myocytes: regulation and consequences of their inhibition.. Cell Signal.

[35] Fujio Y, Nguyen T, Wencker D, Kitsis RN, Walsh K (2000). Akt promotes survival of cardiomyocytes in vitro and protects against ischemia-reperfusion injury in mouse heart.. Circulation.

